# Optimal Spectral Domain Selection for Maximizing Archaeological Signatures: Italy Case Studies

**DOI:** 10.3390/s90301754

**Published:** 2009-03-12

**Authors:** Rosa Maria Cavalli, Simone Pascucci, Stefano Pignatti

**Affiliations:** 1 National Research Council, Institute of Atmospheric Pollution, Via Fosso del Cavaliere, 100, Roma, 00133, Italy; E-Mail: rosa.cavalli@lara.rm.cnr.it; 2 National Research Council, Institute of Methodologies for Environmental Analysis, C.da S. Loja - Zona Industriale, Tito Scalo (PZ), 85050, Italy; E-Mail: pignatti@imaa.cnr.it

**Keywords:** Hyperspectral remote sensing, archaeological spectral features, subsurface structures detection

## Abstract

Different landscape elements, including archaeological remains, can be automatically classified when their spectral characteristics are different, but major difficulties occur when extracting and classifying archaeological spectral features, as archaeological remains do not have unique shape or spectral characteristics. The spectral anomaly characteristics due to buried remains depend strongly on vegetation cover and/or soil types, which can make feature extraction more complicated. For crop areas, such as the test sites selected for this study, soil and moisture changes within near-surface archaeological deposits can influence surface vegetation patterns creating spectral anomalies of various kinds. In this context, this paper analyzes the usefulness of hyperspectral imagery, in the 0.4 to 12.8 μm spectral region, to identify the optimal spectral range for archaeological prospection as a function of the dominant land cover. MIVIS airborne hyperspectral imagery acquired in five different archaeological areas located in Italy has been used. Within these archaeological areas, 97 test sites with homogenous land cover and characterized by a statistically significant number of pixels related to the buried remains have been selected. The archaeological detection potential for all MIVIS bands has been assessed by applying a Separability Index on each spectral anomaly-background system of the test sites. A scatterplot analysis of the SI values vs. the dominant land cover fractional abundances, as retrieved by spectral mixture analysis, was performed to derive the optimal spectral ranges maximizing the archaeological detection. This work demonstrates that whenever we know the dominant land cover fractional abundances in archaeological sites, we can a priori select the optimal spectral range to improve the efficiency of archaeological observations performed by remote sensing data.

## 1. Introduction

As the planet’s exploding human population results in massive developments and changes to the landscape, there is a consequent need for efficient and cost-effective methods to locate, map, and acquire information from sites of our cultural heritage before they are forever lost [1]. Archaeological remote sensing allows large regions to be rapidly investigated for archaeological features [1,2], it can detect features unseen on the surface, precisely map them, and offer interpretations based on their form, distribution, and context [1–3]. Basically, remote sensing can be very useful in preparing an intensive survey campaign or directing fieldwork. In fact, viewing the archaeological structures from ground level generally does not clearly identify the spatial characteristics of these structures or the relationship to the surrounding archaeological sites. The basic assumption of image-interpretation for the recognition of the buried structures is that they can alter the natural trend of the superficial soil and vegetation growth and such alterations can develop into permanent surface spectral features [1,2]. These changes can mark out the pixel appearing with differences, with respect to the adjacent pixels, in color, texture, brightness or combination thereof [4]. The identification of these relevant anomalies, expected in presence of buried man-made structures, depends usually on the experience of the photo-interpreter and his knowledge of the territory [5]. However, environmental factors such as the compaction of soil, moisture content and vegetation impact the effectiveness of the technique to detect subsurface remains [2,5]. In this perspective, one of the challenging research aspects is not only to verify if the most advanced and very high spatial resolution satellite (e.g., IKONOS and QuickBird), or the airborne hyperspectral imagery (e.g., the AHS, the AHI, the CASI and the HyMAP hyperspectral sensors), are feasible for a visual interpretation [6], but it is to identify the image spectral characteristics that bear the highest inherent archaeological information content [7,8]. On the basis of the high spectral and spatial resolution offered by the remotely sensed hyperspectral data, the different spectral anomalies linked to the presence of subsurface archaeological structures should be highlighted by using specific spectral channels and/or their spectral combinations. Recent studies carried out by [9] highlighted the sensitivity of the airborne Multispectral Infrared Visible Imaging Spectrometer (MIVIS) imagery for the detection of surface anomalies linked to the presence of archaeological remains.

In this framework, the paper analyzes the spectral information of MIVIS sensor with respect to the dominant land cover surfacing buried archaeological structures (e.g., stone walls, floors, plaster or tile concentrations, packed earth, pavements near the surface) in 97 test sites (collected within five different archaeological areas in Italy) to assess the best wavelength bands useful for their detection.

Starting from certain training information, i.e. using only those archeological areas where field campaigns and visual interpretation on MIVIS imagery were already performed by archaeologists on not yet excavated buried remnants, 97 pairs of Regions Of Interest (ROI) encompassing the spectral anomaly-background system related to the archaeological remains were manually delineated on MIVIS images. Moreover, to verify the spatial homogeneity of the dominant land cover on the 97 pairs of ROI and to perform a quick test on the spectral orthogonality of the main land cover spectral classes for the further spectral mixture analysis, the Spectral Angle Mapper (SAM) classifier was applied. Spectral mixture analysis was further used to assess the land cover fractional abundances surfacing the buried remains useful for determining whenever the optimal spectral ranges assessed by a Separability Index are connected to the land cover fractional abundances.

## 2. Study Area and Test Sites Selection

**Figure 1.**
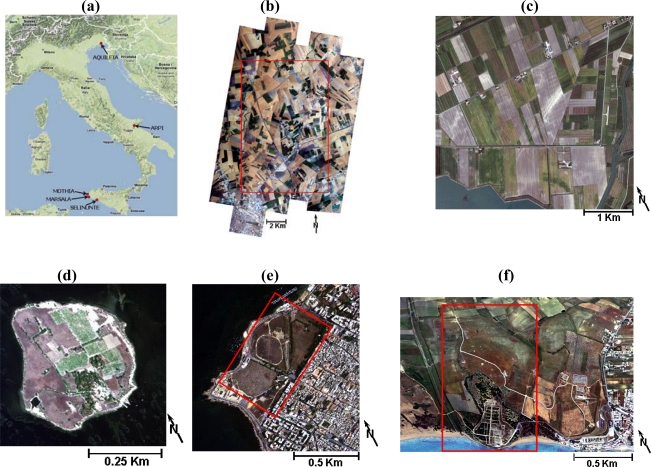
(a) Location of the five study areas over a regional map. (b) MIVIS stripes acquired on the Arpi archaeological area (red box shows the study area), (c) (d) (e) (f) MIVIS images acquired over the Aquileia (resize of 755 × 920 pixels), Mothia (resize of 165 × 165), Marsala (resize of 330 × 330 pixels) and Selinunte (resize of 400 × 920 pixels) study areas, respectively (red box shows the study area).

Five archaeological areas (Figure 1a) were chosen for this study, as they are characterized by not fully excavated subsurface remains, with a sharp geometry, not too deeply buried (i.e., 10–20 cm to 1 m) and with a width and length greater than two MIVIS pixels (3 m/pixel).

The Arpi archaeological area covers an ancient city of Apulia located 8 km NE of the modern city of Foggia in the open countryside and it is considered the metropolis of the ancient Daunia (Figure 1b) [9,10], also known by its Greek place name, Argyripta. Its territory of about 1,000 ha extended to the sea, and Strabo says that from the extent of the city walls one could gather that it had once been one of the greatest cities of Italy. As a protection against the Samnites, Arpi became an ally of Rome (320 BC) and remained faithful until after the battle of Cannae. Arpi enjoyed an economic recovery after the fall of the Roman Empire, but was then destroyed by the Saracens in the eleventh century and, according to medieval sources, it was populace of Arpi who settled nearby Foggia. Excavations begun in the 1940s unearthed the foundations of Hellenic-Roman buildings, some of them boasting lovely mosaic floors. A necropolis was also found, with many graves and small cave burials, with many examples of Apulian vases with their red figures and geometric decorations, dating from between the fourth and third century BC. However, the most important remnants are the ancient city stone walls. For this area, a previous study performed by [9] had identified on MIVIS imagery (Figure 1b) the spectral anomalies relative to the following not yet excavated archaeological structures: an ensemble of features relative to the whole ancient city external perimeter wall (the “aggere”, about 10 Km), two features relative to the defensive structures along the perimeter (i.e. stone walls) and 15 features relative to the main stone streets entering into the ancient metropolis.

The Aquileia archaeological area includes one of the largest and wealthiest cities of the Early Roman Empire that was destroyed by Attila in the mid-5^th^ century. Most of it still lies unexcavated beneath the fields, and as such it constitutes the greatest archaeological reserve of its kind. There are several unexcavated stone walls and ancient roads to be discovered. A previous study carried out in this area by [11] had detected on MIVIS imagery (Figure 1c) features related to 11 ancient villas structures not yet fully excavated.

The Mothia and Marsala archaeological areas cover an ancient Phoenician and an Arab colony founded in Sicily, respectively (Figures 1d,e) [12,13]. The Mothia archaeological area covers an ancient Phoenician colony that was founded at the end of VII century B.C. on the island of San Pantaleo. Thanks to its location, particularly favorable to maritime trade, Mothia soon became one of the most prosperous Western Phoenician colonies. The more outstanding public works date back to the second half of VI century B.C., namely the fortifications, a submerged road that used to link the island to the mainland, near Birgi, the cothon (or drainage basin and harbour) and the main sanctuaries. Mothia is distinguished from all other Phoenician Punic colonies in the Mediterranean area by the conservation status of its urban settlement and by the typology of the architectural structures it contains. Regarding this archeological area, a previous study carried out by [12] had discovered by MIVIS imagery (Figure 1d) 10 linear features related to the ancient street network not yet fully exhumed.

The Marsala archaeological area, at present an Archaeological Park, is characterized by remains of the urban street network, not yet fully excavated, at a small depth. This area was variously investigated by means of excavation tests and air-photo topographic studies. Founded by the Phoenicians under the name of Lilibeo, Marsala lived intensely during the Punic, Roman, Arab and Norman dominations, as a token of which it still keeps important remnants such as pavement stones of ancient streets. As regards the Marsala archeological site, a previous research conducted by [13] had identified on MIVIS imagery (Figure 1e) 14 linear features correlated to the ancient street network not yet fully exhumed.

The Archaeological Park of Selinunte (Figure 1f) is characterized by remains of the urban street network, not yet fully excavated, at a depth of about 50 cm below the ground. Furthermore, the urban street network has already been identified by historical air-photos [14], geophysical surveys and excavation campaigns [15,16]. It is one of the most outstanding cultural heritage sites of Southern Italy, located along the SW coast of Sicily; it was founded in the 7^th^ century BC by colonizers who came from Megara Hyblaea (ancient Greek colony in Sicily). They located the “Acropolis” (public and religious centre of the ancient cities) on a hilly area south of the Manuzza hill. During the two centuries that followed, the city spread eastwards and westwards, along the Cottone and Modione stream valleys, which stretch along the borders of the Manuzza hill [14]. In the Selinunte archeological site, previous studies conducted by [15,16] had identified on MIVIS imagery (Figure 1f) 30 linear features linked to the ancient street network and an ensemble of features related to the city external perimeter wall, both not yet fully exhumed. Moreover, the archaeological areas are all located in agricultural (Arpi and Aquileia) or meadow (Mothia, Marsala and Selinunte) lands in which the main soil type is “soils with carbonates and with clay accumulation”[17].

For these study areas all MIVIS stripes were acquired with similar atmospheric conditions (i.e., clear sky conditions at the acquisition time) and at low solar zenith angle after at least three cloudless days before the acquisitions (i.e. similar soil moisture dryness condition). Moreover, archaeological photo-interpretation and MIVIS features extraction, extensive field work and *in situ* geophysical surveys were carried out by archaeologists [9–16] on the chosen study areas.

On the basis of the archaeological knowledge on the test sites, experts have identified (i.e. manually delineated) on MIVIS imagery spectral anomalies actual related to the buried remnants using also additional information such as the traditional cartography and field surveys. More specifically, only regular pattern anomalies were considered as the presence of geometric features, being quite rare in nature, generally provide useful information for the identification of signs indicating ancient human activities (e.g., ancient stone walls and roads).

According to the abovementioned archaeological areas characteristics’, among the whole spectral anomalies identified on MIVIS imagery in previous studies [9,11–13,15,16], the following 97 test sites were selected: 56 within the Arpi area, 18 inside Aquileia, three for Marsala, three for Mothia, and 17 for Selinunte. For each test site, two ROI, one relative to the spectral anomaly as identified by the archaeologists and one to the spectral anomaly surrounding background, were drawn on the images to be further analyzed by using the SI index. More specifically, the ROI were obtained by manual delineation of the spectral anomalies and their surrounding background primarily on MIVIS RGB (Red-0.70 μm, Green-0.56 μm, and Blue-0.48 μm) and also on peculiar false color composite images. These ROI are characterized by a spatial extent at least of 3 × 10 pixels (i.e. of 90 m^2^) for the spectral anomaly and no less than 5 × 10 pixels for the background.

## 3. Data

### 3.1. Remote sensing data

For this study, MIVIS airborne remote sensing data were processed. The main characteristics of the MIVIS sensor are summarized in Table 1.

The airborne MIVIS imagery acquired from an altitude of 1,500 m a.s.l. (3 m/pixel ground resolution) over Arpi (June 27, 2002, at 10:55 GMT), Aquileia (October 13, 1998, at 11:36 GMT) Marsala (July 12, 2002, at 10:43 GMT), Mothia (May 15, 2002, at 12:06 GMT) and Selinunte (May 23, 1996, at 11:48 GMT) archaeological areas in Italy (see Figure 1) was selected for this study.

### 3.2. Image pre-processing

MIVIS VNIR-SWIR (0.4 to 2.5 μm) data collected over the five archaeological areas was first calibrated to the instrument perceived radiances [15]. Then, to work with comparable datasets, the VNIR-SWIR radiances were corrected to reflectance using the FLAASH module implemented in the ENVI 4.5. software package (ITT; [18]). Whereas, the radiometric calibration of the airborne MIVIS TIR (8.2–12.7 μm) raw data was performed using a two-point calibration technique that is based on the linearity of the detector response over the dynamic range of the instrument [19]. The retrieved pixel TIR spectral radiance (nW cm^−2^ sr^−1^ nm^−1^) is expressed by a linear equation obtained jointing the two reference points (i.e., the maximum and minimum reference values expressed by the radiance value and the corresponding Digital Number).

As regards the atmospheric attenuation of the TIR spectral radiance that includes atmospheric transmission and upwelling atmospheric radiance, the ISAC (In-Scene Atmospheric Compensation) algorithm [20] was employed for the MIVIS TIR atmospheric correction. This algorithm assumes two pixels of the scene to be blackbodies on which neither locations nor temperatures are known. For this study, the “most hits” method as described by [20] was applied and pixels whose emissivity was equal to 1 at the wavelength were used as a marker.

Once the image was atmospherically corrected, the emissivity normalization routine [21,22] implemented in the ENVI 4.5. image processing software and developed by [20–24] was used to retrieve apparent emissivities. This routine, first, derives the brightness temperature of each pixel from the pixel radiance. Next, the apparent emissivity image is obtained by normalizing the radiance of each pixel to the Planck’s curve that is generated from the pixel with the maximum brightness temperature with an emissivity value set to 0.96 (i.e., a reasonable hypothesis for soils).

Final, MIVIS data were geometrically corrected by using an own code, implemented in the IDL 4.5. software package [15,18], which is based on the precise trajectory reconstruction process by using onboard GPS/INS systems and additional ground control point information. In particular, MIVIS data were geocoded using (i) the sensor trajectory (sampled at 1Hz) and the platform attitude (sampled at 25Hz) recorded on board; (ii) the system whiskbroom geometry; (iii) a set of GCPs, extracted from Regional Technical Maps at a scale of 1:10,000, in the navigational data processing to reduce the uncertainties in the trajectory reconstruction. The precise trajectory reconstruction process was used to minimize the effects on the spectral behavior of the pixels due to multiple warping, as the geocoding procedure yielded a RMS error lower than one pixel with a single warping. In fact, MIVIS images yielded a mean RMS error for the selected images less than one pixel so minimizing the effects due to the georegistration error on the location of the detected anomalies.

## 4. Methods

Since buried remains generate slight differences in the spectral characteristics of the overlying terrains, the capability of the high spatial/spectral resolution MIVIS sensor in distinguishing spectral anomalies related to the buried remains was assessed by means of the following methodology: (a) Spectral Angle Mapper (SAM) classification, (b) spectral unmixing (LSU), (c) spectral Separability Index (SI) calculation on a MIVIS per band basis, and (d) scatterplots analysis of the SI vs. land cover fractional abundances (i.e. LSU results).

First, the SAM algorithm was used (a) to perform a quick test on the spectral orthogonality of the land cover spectral classes occurring on the test sites and (b) to verify the homogeneity of the land cover on the anomaly-background system to be further analyzed in a spectral mixture analysis. The SAM supervised classification algorithm was used for several studies, both working in multispectral and hyperspectral data spaces, providing appreciable results [25,26]. The SAM algorithm uses the similarity between the reference class and the pixel spectra by calculating their angular distance in the spectral band space, expressed as a scalar product [25]. SAM input spectra were derived both from Regions Of Interest manually delineated directly on the images, also supported by ASD (portable field spectrometer, 0.35–2.5 μm) field measurements resampled to MIVIS bandpasses.

Second, a constrained Linear Spectral Unmixing procedure [27] (developed in the ENVI 4.5. software package) was trained with the bare soil (“soils with carbonates and with clay accumulation”) and photosynthetic green crop endmembers that depict the land cover variability on the test sites as retrieved by SAM classification. The two endmembers were derived from averaging the ROI spectra selected for training the SAM classification. The LSU, also known as sub-pixel sampling, or spectral mixture analysis, is a widely used procedure to determine the proportion of constituent materials within a pixel based on the materials’ spectral characteristics [27,28]. The LSU procedure assumes that the reflectance of each pixel is a linear combination of endmembers, which are the pure reflectance spectra for each component, thus allowing assessing the percent occurrence of the selected endmembers for each test site. The LSU is analytically expressed as follows [28]:

(1) $$r={\mathrm{Mf}}_{N}+\varepsilon$$

where, *r* is the column vector of the measured radiance/reflectance spectrum with *L* spectral bands, *M* is the *N* × *L* endmember spectra matrix (*N* is the numbers of pure endmembers); *f* is the concentration vector whose components represent the endmember fraction for each endmember, *ε* is the residual error. In this model *M* is the known, while the unknown to be retrieved is the concentration *f_N_*.

Next, the SI spectral index described by [15] was used to rank the capability of detecting the archaeological spectral anomalies by a per-band basis on MIVIS data (i.e. 102 bands). The SI index describes, for the 97 test sites (i.e. corresponding to 97 pairs of ROI encompassing the spectral anomaly-background systems), the tonal differences between the frequency distributions of spectral anomaly pixels and the pixels selected as background for the same spectral anomalies and it is defined as a normalized scalar product expressed as follows:

(2) $$\mathrm{SI}=\left(1-\frac{\int D_{\mathrm{marks}}\;D_{\mathrm{background}}\;\mathrm{dx}}{\sqrt{\int D_{\mathrm{marks}}^{2}\;\mathrm{dx}\int D_{\mathrm{background}}^{2}\mathrm{dx}}}\right)\times 100$$

where, *D_marks_* represents the frequency distribution of the digital values of those pixels belonging to the archaeological spectral anomalies in all images, while *D_background_* corresponds to the frequency distribution of those pixels selected as background. According to the index definition, the SI is a parameter related to the brightness similarity of the spectral anomalies with respect to the background.

The final step of the proposed procedure refers to the scatterplot analysis of the SI values with respect to the spectral unmixing results (i.e. the endmembers fractional abundance) of the anomaly-background systems. More specifically, the scatterplot of the SI values (calculated for each MIVIS band) vs. the fractional abundances of the two endmembers was used to highlight the spectral ranges maximizing the archaeological signatures.

## 5. Results and Discussion

As the spectral anomalies characteristics due to buried remains depend on vegetation cover and/or soil types, the SAM algorithm was applied to MIVIS images to verify the land cover occurring on the anomaly-background system of the 97 known archaeological anomalies. The following classes were used to train the SAM classifier for all the archaeological areas: artificial surfaces, forest areas, water bodies, bare soil, photosynthetic green crop and dry vegetation (e.g. stubs, i.e. a combination of crop residues and soil). The SAM results show that the 97 anomalies-background systems are characterized by homogeneous land cover and, therefore, they satisfy the Separability Index calculation requirements (i.e. anomaly and background have to be on the same land cover).

**Figure 2.**
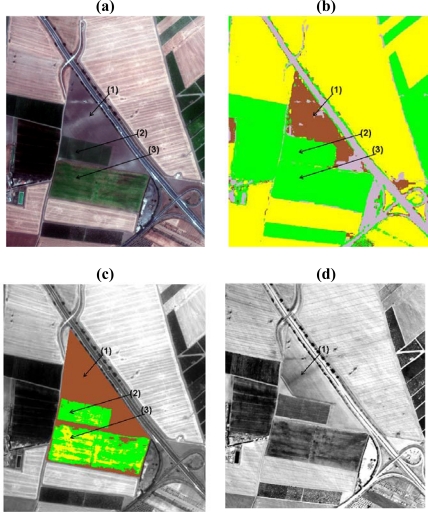
(a) Example of three test sites with the archaeological spectral anomalies (black arrow) detected on MIVIS imagery of Arpi (Italy). (b) SAM results for the same area (the yellow, green, grey, maroon colors depict the dry vegetation, photosynthetic green crop, artificial surfaces and bare soil, respectively). (c) LSU results for the three test sites (the brown color depicts the anomaly(1)-background system covered by more than 75 % of bare soil; the green color depicts the anomaly (2)-background system covered by more than 75 % of photosynthetic green crop; the yellow color shows the anomaly(3)-background system covered by a mixture of bare soil and green crop endmembers ranging between 25 % and 75 %). (d) MIVIS TIR image for the same test sites (MIVIS bands 93 only for visualization purposes).

For example, Figure 2b shows the SAM classification attained for three spectral anomalies in the Arpi archaeological area. However, looking at the SAM classification of Figure 2b, it can be noted that the spectral anomalies #2 and #3 are classified by the same land cover (i.e. green crop), even though they are characterized by two different crop densities. Therefore, a properly supervised classification of the five archaeological areas should imply the use of a higher thematic level, which will be therefore site-specific and not useful for identifying the optimal spectral range for archaeological prospection at a broad-scale (i.e. encompassing a wide range of anomaly-background systems), such as the aim of this study.

This lack of accuracy depicted by the SAM classification for a broad-scale of anomaly-background systems was solved by the use of the “constrain” LSU procedure [27] as based on endmembers and not on specific land covers. Therefore, bare soil and photosynthetic green crop were applied as endmembers in the LSU procedure by constraining the fractions to sum to one. In particular, the two spectral endmembers were derived by averaging the ASD field spectra acquired on the two dominant land cover in each selected test site and resampled to MIVIS bandpasses. Moreover, the “shade endmember” was not included in each endmember model to account for variation in illumination, as the 97 anomaly-background systems were not significantly affected by shadowing.

Figure 2c shows how the LSU procedure allows depicting the actual crop/soil distribution characteristics and abundance in the test sites. This confirms that the LSU can be applicable to describe the wide range of the anomaly-background systems used for this study.

**Figure 3.**
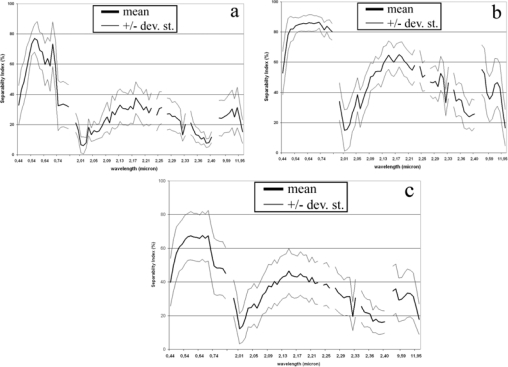
The graphs show the SI trend for the different MIVIS wavelength bands (VNIR, SWIR and TIR spectral regions) for all the test sites showing more than 75 % (from the unmixing results) of (a) green crop and (b) bare soil endmembers. Graph 4c shows the SI behaviour of a mixture of bare soil and green crop endmembers.

The final step of the proposed procedure refers to the scatterplot analysis of the SI values with respect to the spectral unmixing results. More specifically, the scatterplot of the SI values (calculated for each MIVIS band) vs. the fractional abundances of the two endmembers was used to highlight the spectral ranges maximizing the archaeological signatures. By jointly analyzing the scatterplots attained for the SI values (attained by calculating the index for all the MIVIS wavelength bands) vs. the LSU results (i.e. the endmembers fractional abundance for the anomaly-background systems), the most promising spectral regions in terms of archaeological detection potential in relation to the dominant land cover type were assessed. The first outcome of the scatterplot analysis stresses that the SI trend with respect to the wavelength bands deeply varies in function of the abundance of the two occurring land covers (Figure 3). In the case that the subsurface structures are covered by more than 75 % of photosynthetic green crop (i.e. actual percent cover of green crop vegetation), the 0.50 to 0.76 μm (Visible-Near Infrared) spectral range resulted to be, from the SI values (Figure 3a), the most suitable for their detection. In particular, Figure 3a highlights that if the buried structures are covered by more than 75 % of green crop vegetation, i.e. in 12 test sites as derived from the LSU results (see e.g. Figure 2c test site (2) depicted in green color), the two most promising wavelengths for their detection are the chlorophyll peak at 0.56 μm (Visible region) and the red edge region (0.67 to 0.72 μm; NIR region), as their SI values are the highest among all MIVIS wavelength bands. This result confirms that the variation induced by the subsurface structures (i.e., stone walls, floors, plaster or tile concentrations, packed earth, pavements near the surface) to the natural vegetation growth and / or appearances (i.e., foliage chlorosis depicting different plant stress factors) is primarily detectable by the 0.56 μm and the 0.67 to 0.72 μm wavelengths, which are sensitive for the vegetation stress detection [27,28] (Figure 2a).

Whereas, looking at the SI values for those sites where bare soil covers the archaeological structures for more than 75 %, i.e. in 13 test sites as identified by LSU results [e.g. Figure 2c test site (1) depicted in green color], the 0.48 to 0.82 μm (VNIR) spectral range is the most suitable to stress the anomalies related to the buried structures (Figure 3b). Moreover, Figure 3b shows that if bare soil covers up the buried structures for more than 75 %, MIVIS SWIR and TIR spectral regions, appear less performing in the discrimination between the spectral anomalies and background. In fact, only in those test sites where the buried structures are covered by bare soils, the 2.1 to 2.2 μm (SWIR) and the 8.3 to 9.1 μm (TIR) spectral ranges supply high SI values so allowing a more clear detection of the buried structures.

The results attained for the TIR range [e.g. Figure 3d, test site (1)] for all the test sites highlight that the heat transfer through the soil is affected by the presence of buried objects; therefore, the material inertial resistance to temperature fluctuations (i.e. the buried material thermal inertia; [8]) can be useful for detecting the subsurface archaeological structures.

Instead, if above the subsurface remnants a mixture of bare soil and green crop endmembers (ranging between 25 % and 75 %), i.e. in 72 test sites as identified by the LSU results [e.g. Figure 2c test site (3), depicted in yellow color], the MIVIS bands maximizing their detection potential show lower SI mean values and a higher variability (standard deviation) for all the bands with respect to the green crop (> 75 %) and bare soil (> 75 %) SI values (Figures 3a,b). Looking at Figure 3c it is possible to observe a behavior, in terms of SI mean values, which could be viewed as a combination of the green crop and bare soil SI trend values for the whole MIVIS spectral ranges (VNIR-SWIR-TIR).

As final remark, from the analysis of the graphs in Figure 3 it can be point out that an hyperspectral sensor covering the 0.4–0.8 μm spectral range (VNIR) with a high spectral resolution (0.1–0.3 μm) can be adequate for archaeological investigations aimed at maximizing the spectral anomalies to detect the buried archaeological structures (e.g. Figure 3) whenever the surfacing land cover abundances are known. To summarize, the results attained for the MIVIS data in this study have to be considered in view of both the sensors’ characteristics/sensitivity and the atmospheric conditions at the time and season (e.g., the distance from the last rainy day) of acquisition.

## 6. Conclusions

This study demonstrates that the combined use of the Separability Index and the land cover fractional abundance (as derived by spectral unmixing) is a powerful technique to identify the sensors’ bands most profitable for the archaeological prospection.

The results attained for the selected test areas reveal that if the dominant land cover abundances in agricultural land areas are known, we can a priori select the optimal spectral range for remote sensing data suitable to the enhancement of spectral anomalies related to the subsurface archaeological remains. Furthermore, the results show that high spatial resolution VNIR multispectral data can be extremely effective for the analysis of large cultural heritage assets. Noteworthy is also the usefulness of the TIR spectral range where archaeological structures are covered by bare soils.

Further research will include the evaluation of the efficiency and robustness of the proposed procedure for different airborne hyperspectral imagery and time and season of acquisition and soil moisture conditions in the same archaeological sites and including more endmembers in the unmixing procedure. This will allow to develop a quick and affordable tool for archaeologists whenever starting their analysis on airborne remote sensing data with scarce information. However, the relatively high costs of high spatial/spectral remote sensing data must be balanced against the real larger costs of planning decisions based on a poor knowledge of what lies in the subsurface and of failing to correctly locate archaeological features and other culturally sensitive deposits prior to their disturbance.

## Figures and Tables

**Table 1.** Characteristics of the MIVIS sensor used for this study.

	**Spectral Region**	**Spectral Resolution (μm)**	**Spectral Range (μm)**	**SNR**	**Spatial Resolution (m)**	**IFOV (deg)**	**Swath width**
**MIVIS**	VNIR (28 ch.)	0.02 (VIS)	0.43–0.83 (VIS)	<400	≅ 3 (at 1,500 m flight altitude)	0.115	4.2 km at 3,000 m of (relative) flight height
		0.05 (NIR)	1.15–1.55 (NIR)	<600			
	SWIR (64 ch.)	0.09	1.983–2.478	<200			
	TIR (10 ch.)	0.34–0.54	8.180–12.700	<700			
